# Factors influencing front line treatment of chronic lymphocytic leukemia: A French real‐world study

**DOI:** 10.1002/cncr.70406

**Published:** 2026-04-15

**Authors:** Arthur Coste, Nanthara Sritharan, Anne Lok, Cécile Tomowiak, Emmanuelle Ferrant, Aline Clavert, Damien Roos‐Weil, Florian Bouclet, Adrien Caillet, Carolyne Croizier, Nicolas Stocker, Emmanuelle Tchernonog, Pierre Feugier, Alberto Santagostino, Bénédicte Hivert, Anne‐Sophie Michallet, Sophie De Guibert, Diane Lara, Agathe Waultier‐Rascalou, Fatiha Merabet, Amandine Durand, Lucile Bussot, Kamel Laribi, Marie‐sarah Dilhuydy, Vincent Lévy, Anne Quinquenel

**Affiliations:** ^1^ Université Reims Champagne‐Ardenne Hématologie clinique CHU de Reims Reims France; ^2^ Département de Recherche Clinique Hôpital Avicenne AP‐HP Université Sorbonne Paris Nord Bobigny France; ^3^ Hématologie clinique CHU de Nantes Nantes France; ^4^ Hématologie clinique CHU de Poitiers Poitiers France; ^5^ Hématologie clinique Groupement Hospitalier Lyon Sud Hospices Civiles de Lyon Lyon France; ^6^ Hématologie clinique CHU d’Angers Angers France; ^7^ Sorbonne Université Hématologie clinique Hôpital de la Pitié salpêtrière AP‐HP Paris France; ^8^ Hématologie clinique Centre Henri‐Becquerel Rouen France; ^9^ Hématologie clinique CHU Jean Minjoz Besançon France; ^10^ Hématologie clinique CHU Estaing Clermont‐Ferrand France; ^11^ Hématologie clinique et thérapie cellulaire Hôpital Saint Antoine AP‐HP Paris France; ^12^ Hématologie clinique CHRU de Montpellier Montpellier France; ^13^ Hématologie clinique CHRU de Nancy Vandœuvre‐lès‐Nancy France; ^14^ Hématologie clinique CH de Troyes Troyes France; ^15^ Hématologie clinique Hôpital Saint Vincent de Paul Lille France; ^16^ Hématologie clinique Centre Léon Berard Lyon France; ^17^ Hématologie clinique CHU de Rennes‐Pontchaillou Rennes France; ^18^ Hématologie clinique CH de Libourne Libourne France; ^19^ Hématologie clinique CHU de Nîmes Nîmes France; ^20^ Hématologie clinique CH de Versailles Versailles France; ^21^ Hématologie clinque CHU de Dijon Dijon France; ^22^ Hématologie clinque CHU de Grenoble Grenoble France; ^23^ Hématologie clinique CH Le Mans Le Mans France; ^24^ Hématologie clinique CHU de Bordeaux Pessac France; ^25^ Université Reims Champagne‐Ardenne, UR 7509‐IRMAIC hématologie clinique, CHU de Reims Reims France

**Keywords:** chronic lymphocytic leukemia, first‐line treatment, real‐world evidence, physician decisions

## Abstract

**Background:**

Real‐world data are an essential complement to clinical trials. This is particularly true for chronic lymphocytic leukemia, where five first‐line options have never been directly compared.

**Methods:**

The authors present the results of a national multicenter real‐world study focusing on treatment choices in frontline chronic lymphocytic leukemia (CLL) and the criteria underlying this choice. Patients’ medical records were included over a 6‐month period in 25 centers.

**Results:**

The majority of patients received obinutuzumab and venetoclax, especially those with mutated *IGHV* status. Patients harboring *TP53* alterations were almost all treated with Bruton tyrosine kinase inhibitors, with a preference for zanubrutinib. Patients initiated on continuous Bruton tyrosine kinase inhibitors (BTKi) regimens were significantly older; second‐generation BTKi, acalabrutinib and zanubrutinib were mostly prescribed. The most cited choice criteria by physicians were genetic prognostic factors, followed by fixed treatment duration and patient logistics considerations. Multiple correspondence analysis and unsupervised hierarchical clustering analysis allowed to identify two distinct patient profiles: younger patients, mostly with mutated *IGHV* status, who were mainly treated with combined drug regimens due to their fixed duration, and older patients, largely treated with BTKi because of the possibility of outpatient management.

**Conclusion:**

This study is the first to report real‐world evidence on treatment choice in first‐line CLL and highlight two distinct groups of patients.

## Abstract 2

This work presents factors influencing treating physicians when choosing first line chronic lymphocytic leukemia treatment.

## INTRODUCTION

In recent years, there has been increasing interest in real‐world data (RWD). This has led to the development of real‐world evidence (RWE) medicine based on the usage and potential benefits or risks of a medical product derived from the analysis of RWD.^1^ RWE can help address clinically relevant questions that are not explored in clinical trials, particularly when dealing with patients with associated medications and organ dysfunction.

The generation of RWE data is particularly important in chronic lymphocytic leukemia (CLL). Indeed, in France, five chemo‐free first‐line therapeutic options are currently reimbursed, including the first‐ and second‐generation Bruton tyrosine kinase inhibitors (BTKi) ibrutinib, acalabrutinib with or without obinutuzumab, zanubrutinib administered until progression or unacceptable toxicity,^2^, ^3^, ^4^, ^5^, ^6^ and fixed‐duration combination regimens of obinutuzumab‐venetoclax (O‐Ven) and venetoclax‐ibrutinib (Ven‐I).^7^, ^8^, ^9^ All these treatments have shown superiority over reference chemoimmunotherapy regimens but have never been directly compared in clinical trials for treatment‐naive patients. Treatment guidelines are available in many countries and rely on both scientific data and expert opinion.^10^, ^11^, ^12^, ^13^ Thus, although all recommendations clearly advocate the use of BTKi in patients with *TP53* abnormalities and the combination of obinutuzumab and venetoclax in patients with mutated *IGHV* status, all available treatments can be used in patients with unmutated *IGHV* status, without it being possible to favor one treatment over the other based on biological criteria alone. In this situation, treatment decisions are often influenced by comorbidities, co‐medications, disease presentation, and patient preferences.

In this unusual context of multiple available and fully reimbursed options, RWD evaluating treatment choices and underlying reasons in patients with frontline CLL are very limited. In France, the initiation of antineoplastic treatment must be systematically validated in a multidisciplinary consultation meeting. Leveraging this national obligation, we designed a national study aimed at analyzing not only the geographical distribution of first‐line treatments but also at evaluating the criteria considered important by clinicians in justifying these choices.

## MATERIALS AND METHODS

### Study design and population

All centers of the French Innovative Leukemia Organization (FILO) were invited to participate in this multicenter, observational study conducted across France.^14^

Patients were eligible if they were over 18 years of age, had previously untreated CLL, and required frontline treatment. Consent for inclusion in the study was obtained from the referring physicians. The exclusion criteria were as follows: (1) patient refusal to participate, (2) watch‐and‐wait strategy, and (3) Richter transformation.

For each first‐line CLL patient discussed during a multidisciplinary consultation, a questionnaire was completed by the referring physician immediately following the discussion. This form collected anonymized data on the clinicobiological characteristics of the patient's CLL, the chosen treatment, and the criteria that motivated the choice (Figure S1).

This noninterventional study was conducted in accordance with the French regulations and the Declaration of Helsinki. Informed consent was obtained from all patients before data collection.

### Collected data

This questionnaire allowed for quick and efficient completion during multidisciplinary consultation meetings. The following information was collected: geographical location and type of center (university or general hospital, private hospital clinic, or cancer center), patient age and sex, tumor or cytopenic presentation, available prognostic factors (cytogenetic: *11q* deletion, *17p* deletion, and complex karyotype [five or more abnormalities]; molecular: *TP53* abnormality and *IGHV* mutational status), validated treatment (ibrutinib, acalabrutinib ± obinutuzumab, zanubrutinib, obinutuzumab + venetoclax, ibrutinib venetoclax, immunochemotherapy or clinical trial), and the criteria on which the decision was based (comorbidities, concomitant medications, infectious history, prognostic factors, management logistics, fixed duration, and prescriber experience). *TP53* abnormality, 17p deletion, and complex karyotype were considered as high risks genetic abnormalities.

All anonymized forms were then collected, and all available data were stored online in a dedicated, secure health care database (webtrial from Quantic‐soft).

### Statistical analysis

Descriptive statistics were used to summarize the collected data. Categorical variables are presented as frequencies and percentages, and continuous variables are described using the median and interquartile range (IQR).

Logistic regression models were used to assess the association between patient characteristics and treatment choice. Results are reported as odds ratios (ORs) with corresponding 95% confidence intervals (95% CIs). A multivariate logistic regression analysis was performed on significant variables, while respecting an appropriate number of events per variable to avoid overfitting the model.

To explore the criteria influencing treatment decisions, multiple correspondence analyses (MCA)^15^ was performed. The MCA included all variables corresponding to the therapeutic choice criteria reported by physicians, as well as the treatment actually chosen. Unsupervised hierarchical clustering analysis (HCA) was then applied to the MCA results to identify clusters. Clustering was based on the Euclidean distance and Ward’s method. The optimal number of clusters was determined by identifying the local maximum in inertia gain while minimizing the number of clusters to ensure maximum inter‐group variance and minimum intragroup variance. To verify the robustness of the cluster number, *k*‐means clustering^16^ was applied using the same number of clusters identified in the HCA.

To characterize individual clusters, variables were compared using Fisher exact test or χ^2^ for categorical data and the Mann‐Whitney *U* test for continuous data.

No imputation method was applied for missing data, as only one patient was missing in the MCA and clustering analyses.

All statistical analyses were performed using R software (version 3.6.1).^17^ Statistical significance was set at *p* < .05.

## RESULTS

Given the small number of patients treated with ibrutinib or immunotherapy solely (primarily CD20 antibodies in case of autoimmune anemia), these two groups were excluded from the statistical analysis because they would not allow robust statistical inference or meaningful pattern identification. Similarly, patients included in clinical trials were excluded to better reflect real‐world clinical practices.

### Demographics

**FIGURE 1:**
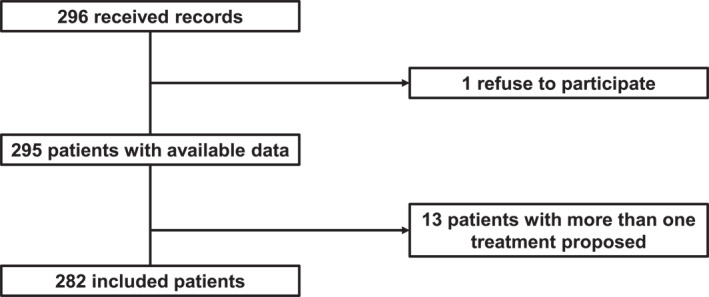
Flowchart of included patients.

Between February and September 2024, a total of 295 frontline treatment records were collected from 25 participating centers. After excluding one patient who declined participation and 12 patients for whom more than one treatment option was selected in the questionnaire, 282 patient records were included in the final analysis (Figure 1).

Among the 282 patients included, 61.6% were male and 38.4% were female. The median age at the time of treatment decision was 72 years (IQR, 63.8–79.0), with 6.4% (*n* = 18) of patients 50 years old or younger. The majority of patients (71.8%, *n* = 196) had a tumoral form of the disease, whereas 55.1% (*n* = 147) had cytopenias.

*TP53* or del 17p exploration data were available for 93.6% of patients, and *IGHV* status was known for 81.5% before therapeutic decision. Karyotype evaluation was only available for 63% of patients. Among included patients, 102 (44.3%) had a mutated *IGHV* status (M‐*IGHV*) and 41 (18.0%) had an 11q deletion. Seventeen patients (6.9%) harbored a 17p deletion, 32 (12.1%) had a *TP53* mutation, and 14 (7.9%) had a complex karyotype (defined as five or more chromosomal abnormalities) (Table 1).

**TABLE 1:** Patient characteristics.

Included patients	282
Median age [IQR], years	72.0 [63.8–79.0]
Sex (male), No. (%)	173 (61.6)
Oncology meeting center, No. (%)	
University hospital	234 (83)
General hospital	26 (9.2)
Cancer center	21 (7.4)
Clinical and biological data, No. (%)	
Tumoral form	196 (71.8)
Cytopenia	147 (55.1)
Genetic abnormalities, No. (%)	
Del 17p	17 (6.9)
*TP53* mutation	27 (10.6)
Del 17p and/or *TP53* mutation	32 (12.1)
Mutated *IGHV* status	102 (44.3)
Complex karyotype	14 (7.9)
Del 11q	41 (18)

Abbreviation: IQR, interquartile range.

### Treatment choice

**FIGURE 2:**
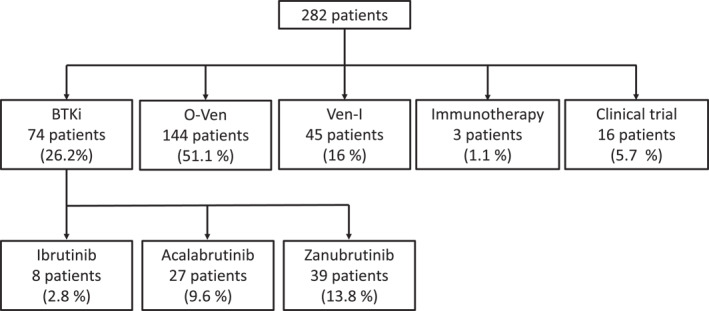
Chosen first‐line treatments. BTKi, Bruton Tyrosine Kinase inhibitor; O‐Ven, obinutuzumab and venetoclax; Ven‐I, venetoclax and ibrutinib.

Most patients (*n* = 144, 51.1%) received a 12‐month combined regimen of obinutuzumab and venetoclax (O‐Ven). BTKis were prescribed to 74 patients (26.2%). Among these, 39 patients (13.8%) received zanubrutinib, representing 52.7% of BTKi‐treated patients, 27 patients received acalabrutinib (9.8% of the total cohort; 36% of BTKi‐treated patients), and only eight patients (2.8% of the total cohort; 10.8% of BTKi‐treated patients) received ibrutinib. The 12‐month combined regimen of venetoclax and ibrutinib (Ven‐I) was selected for 45 patients (16.0%). Sixteen patients (5.7%) were enrolled in clinical trials. Notably, no patient received immunochemotherapy, and three patients with autoimmune hemolytic anemia were treated with immunotherapy solely. Figure 2 shows the reported treatment choices.

### Association between patient and CLL characteristics and treatment choices

As shown in Table 2, univariate logistic regression analyses identified several factors that were significantly associated with treatment choice. Age emerged as a key determinant: older patients were more likely to receive BTK inhibitors, including acalabrutinib (OR, 1.1; 95% CI, 1.1–1.2, *p* = .0003) and zanubrutinib (OR, 1.1; 95% CI, 1.0–1.1, *p* < .0001). Conversely, the combination regimen of venetoclax and ibrutinib was preferentially used in younger patients (OR, 0.9; 95% CI, 0.9–0.9, *p* < .0001).

**TABLE 2:** Results of the univariate logistic regression analysis.

	Acalabrutinib			Zanubrutinib			O‐Ven			Ven‐I		
Variable	OR (95% CI)	*p*	*N*	OR (95% CI)	*p*	*N*	OR (95% CI)	*p*	*N*	OR (95% CI)	*p*	*N*
Age	1.10 (1.05–1.15)	.0003	280	1.08 (1.04–1.13)	<.0001	280	0.99 (0.97–1.01)	.36	279	0.91 (0.89–0.94)	<.0001	279
Sex (male)	1.07 (0.48–2.51)	.88	281	1.33(0.63–2.99)	.47	281	0.84 (0.50–1.40)	.5	280	0.98 (0.50–1.20)	.9	280
Oncology meeting and treatment place	2.74 (1.01–9.58)	.07	281	1.33(0.64–2.91)	.46	281	0.91 (0.55–1.49)	.7	280	0.8 (0.42–1.58)	.51	280
Tumoral form	0.77 (0.32–1.97)	.56	273	0.72 (0.35–1.53)	38	273	0.74 (0.43–1.26)	.27	272	4.62 (1.78–15.8)	.005	272
Cytopenia	1.97 (0.81–5.29)	.15	267	1.93 (0.95–4.15)	.08	267	0.95 (0.59–1.54)	.84	266	0.47 (0.24–0.90)	.03	266
Del 17p	1.21 (0.18–4.65)	.81	248	5.77 (1.94–16.42)	.001	248	0.05 (0.003–0.25)	.004	248	0.3 (0.02–1.52)	.25	247
*TP53* mutation	1.81 (0.5–5.30)	.32	255	14.58 (6.0–36.67)	<.0001	255	0.03 (0.001–0.13)	.0004	255	0.17 (0.01–0.86)	.09	254
Mutated *IGHV*	0.56 (0.22–1.31)	.19	230	0.79 (0.34–1.75)	.57	230	3.87 (2.24–6.81)	<.0001	230	0.21 (0.08–0.48)	.0005	229
Complex karyotype	0.52 (0.03–2.83)	.54	177	3.22 (0.82–10.79)	.07	177	0.49 (0.14–1.47)	.21	177	0.33 (0.02–1.73)	.29	176
Del 11q	0.90 (0.25–2.56)	.86	280	1.43 (0.5–3.64)	.47	228	0.5 (0.25–0.99)	.0049	228	2.55 (1.13–5.55)	.02	227

Abbreviations: CI, confidence interval; OR, odds ratio; O‐Ven, obinutuzumab‐venetoclax; Ven‐I, venetoclax‐ibrutinib.

The presence of high‐risk genetic abnormalities was a strong predictor of BTKi use. In particular, the choice of zanubrutinib was significantly associated with the presence of 17p deletion (OR, 5.8; 95% CI, 1.9–16.4, *p* = .001) and/or *TP53* mutation (OR, 14.6; 95% CI, 6.0–36.7, *p* < .0001).

The use of O‐Ven treatment was significantly more frequent in patients with M‐*IGHV* status (OR, 3.8; 95% CI, 2.2–6.8, *p* < .0001). Conversely, O‐Ven was significantly underprescribed in the presence of high‐risk cytogenetic features, such as 17p deletion (OR, 0.1; 95% CI, 0.003–0.3, *p* = .004) and/or *TP53* mutation (OR, 0.03; 95% CI, 0.001–0.1, *p* = .0004).

The prescription of the combination of ibrutinib and venetoclax decreased with increasing age (OR, 0.9; 95% CI, 0.9–0.9, *p* < .001). Furthermore, a clinical tumor form of the disease was associated with an increased prescription in univariate analysis only (OR, 4.6; 95% CI, 1.8–15.8, *p* = .005).

Multivariate logistic regression confirmed the previous observations (Table 3).

**TABLE 3:** Results of the multivariate logistic regression analysis on significant univariate variables.

	Acalabrutinib			Zanubrutinib			O‐Ven			Ven‐I		
Variable	OR (95% CI)	*p*	*N*	OR (95% CI)	*p*	*N*	OR (95% CI)	*p*	*N*	OR (95% CI)	*p*	*N*
Age	1.1 (1.06–1.15)	<.0001	279	1.08 (1.04–1.14)	.001	262				0.91 (0.87–0.94)	<.0001	227
Oncology meeting and treatment place	3.9 (1.38–1.43)	.02	279									
*TP53* or del 17p				14.21 (5.83–36.32)	<.0001	262	0.06 (0.01–0.23)	.0003	203			
Mutated *IGHV*							4.37 (2.3–8.61)	<.0001	203	0.22 (0.08–0.53)	.001	227
Del 11q							0.55 (0.25–1.20)	.14	203			

Abbreviations: CI, confidence interval; OR, odds ratio; O‐Ven, obinutuzumab‐venetoclax; Ven‐I, venetoclax‐ibrutinib.

### Factors influencing treatment choice by physicians

As shown in Table 4, the most frequent factors influencing physicians’ treatment choices were the presence of prognostic markers (59.8%) and the preference for fixed‐duration treatment (49.8%). Comorbidities were reported to influence treatment decisions in 29.5% of cases, with cardiovascular comorbidities being the most common (18.1%), followed by chronic hypertension and diabetes. When clinically feasible, patient preferences were also considered, particularly regarding ambulatory treatment with oral agents only (BTKi alone or with venetoclax). Additionally, the medical doctor’s experience with some treatments or inexperience with others also played an important role in the treatment choice.

**TABLE 4:** Factors influencing treatment decisions by physicians.

Chosen criteria	No. (%)	No. total
Cardiac dysfunction	51 (18.1)	281
Kidney dysfunction	8 (2.8)	281
Other comorbidity	24 (8.6)	280
Anticoagulant or antiplatelets	23 (8.2)	281
Other medication	2 (0.7)	281
History of severe infection	4 (1.4)	281
Patient’s prognostic factors	168 (59.8)	281
Patient’s logistic	75 (26.7)	281
Ambulatory management	20 (7.1)	281
Fixed duration	140 (49.8)	281
Practitioner experience	49 (17.4)	281

### Multiple correspondence analyses

MCA was performed on the most influential decision criteria for the first‐line treatment choice (Figure S2).

The first dimension (20.9% of inertia) was primarily driven by the use of BTK inhibitors, particularly acalabrutinib and zanubrutinib, and was associated with the absence of cardiac comorbidities and anticoagulant therapy. Additionally, patients' willingness to undergo ambulatory treatment also appears to be a driving factor.

The second dimension (19.4%) was largely explained by the presence of renal comorbidities, use of antiaggregant or anticoagulant agents, and the choice of Ven‐I combination therapy, highlighting the influence of the overall patient condition and treatment feasibility.

MCA projections revealed distinct clinical profiles based on treatment strategies. Patients treated with BTKi‐based regimens generally lacked cardiac comorbidities and antithrombotic treatment but often had renal dysfunction and required outpatient care. The O‐Ven regimen was preferred for patients without renal dysfunction but with cardiac comorbidities and those receiving anticoagulant or antiaggregant therapy. The Ven‐I regimen profile was more heterogeneous but shared similarities with that of the O‐Ven group.

### Hierarchical clustering analysis and description of the two clusters identified

**FIGURE 3:**
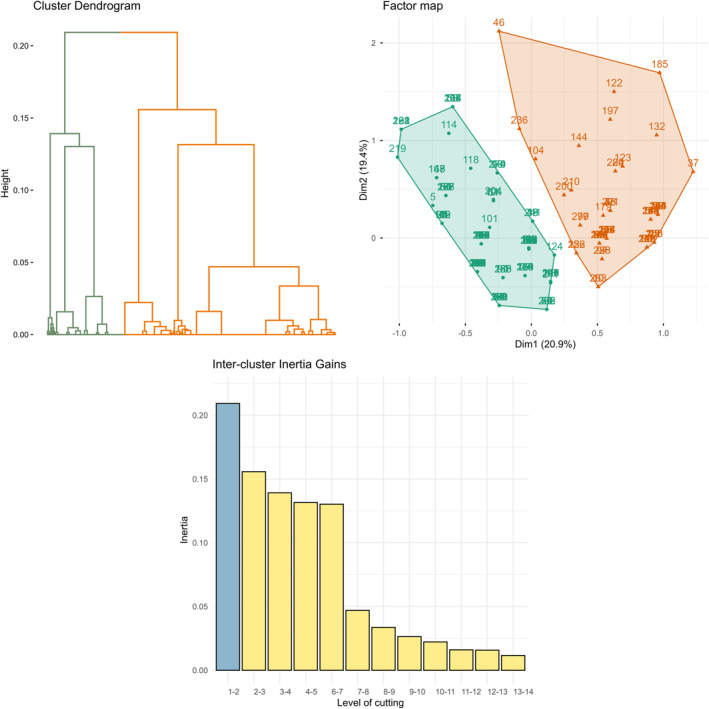
Patient clustering from multiple correspondence analyses: dendrogram, clustered individuals on the factorial plan, and inertia on the first dimensions.

Hierarchical clustering was performed using the first six MCA dimensions, which explained 83% of the overall variance and identified two distinct patient clusters (Figure 3).

The first cluster (*n* = 175) consisted predominantly of younger patients (median age, 69 [61–76] years) than those in cluster 2 (median age, 78 [72–84] years; *p* < .001). This group exhibited significantly fewer high‐risk genetic abnormalities, including a markedly lower frequency of *TP53* mutations (0.6% vs. 30.3%, *p* < .001) and 17p deletion (0.6% vs. 14.9%, *p* < .001), and a higher proportion of mutated *IGHV* status (50.6% vs. 33.9%, *p* = .04). Most patients received fixed‐duration O‐Ven treatment (77.1% vs. 10.1%, *p* < .001), with treatment choice primarily driven by the intent for a time‐limited approach. This cluster also had significantly fewer logistical challenges (16.6% vs. 57.0%, *p* < .001) and no cases of renal impairment (0.0% vs. 8.9%, *p* < .001).

The second cluster (*n* = 79) included significantly older patients and a higher frequency of adverse genetic features, such as *TP53* mutations and 17p deletions (*p* < .001 for both, compared to cluster 1). These patients were mainly treated with BTKi, particularly zanubrutinib (49.4%) and acalabrutinib (34.2%), indicating a preference for continuous oral therapies. In this group, outpatient management and the presence of renal dysfunction (*p* < .001 for both) were crucial factors influencing treatment decisions, suggesting that treatment feasibility in an ambulatory setting was a key driver of therapeutic decisions (Table 5).

**TABLE 5:** Cluster characteristics identified via hierarchical clustering.

	Cluster 1, *N* = 175 patients	Cluster 2, *N* = 79 patients	*p*	*N*
Median age [IQR], years	69.0 [61.0–76.0]	78.0 [72.0–84.0]	<.001	
Sex (male), No. (%)	108 (61.7)	48 (61.5)	>.9	
Clinical/biological data, No. (%)				
Tumoral form	128 (74.4)	49 (66.2)	.25	246
Cytopenia	88 (52.1)	47 (64.4)	.10	242
Genetic abnormalities, No. (%)				
Del 17p	1 (0.6)	10 (14.9)	<.001	227
Mutated *TP53*	1 (0.6)	20 (30.3)	<.001	234
Del 17p and/or mutated *TP53*	2 (1.2)	22 (31.0)	<.001	241
Mutated *IGHV*	78 (50.6)	20 (33.9)	.04	213
Complex karyotype	6 (5.2)	5 (9.8)	.31	167
Del 11q	26 (17.3)	12 (20.7)	.72	208
Chosen treatment, No. (%)			<.001	254
Acalabrutinib	0 (0.0)	27 (34.2)		
Zanubrutinib	0 (0.0)	39 (49.4)		
O‐Ven	135 (77.1)	8 (10.1)		
Ven‐I	40 (22.9)	5 (6.3)		
Criteria, No. (%)				254
Cardiac condition	38 (21.7)	9 (11.4)	.07	
Kidney dysfunction	0 (0.0)	7 (8.9)	<.001	
Other comorbidity	13 (7.4)	8 (10.1)	.63	
Anticoagulant or antiplatelets	17 (9.7)	4 (5.1)	.32	
History of severe infection	1 (0.6)	3 (3.8)	.09	
Patient’s prognostic factors	108 (61.7)	41 (51.9)	.18	
Patient’s logistic	28 (16.0)	41 (51.9)	<.001	
Ambulatory management	6 (3.4)	10 (12.7)	.01	
Fixed duration	133 (76.0)	0 (0.0)	<.001	
Practitioner experience	31 (17.7)	31 (17.7)	>.9	

Abbreviations: IQR, interquartile range; O‐Ven, obinutuzumab‐venetoclax; Ven‐I, venetoclax‐ibrutinib.

Overall, this clustering approach highlights two contrasting therapeutic profiles: one oriented toward younger, lower‐risk patients favoring time‐limited, intensive regimens and the other toward older, higher‐risk patients for whom continuous oral therapies offer a more practical and tolerable alternative.

A descriptive analysis grouping centers by type (academic vs. nonacademic) showed similar distributions of patients across clusters and comparable treatment patterns, suggesting that therapeutic decisions were consistent across different center types (Table S3).

## DISCUSSION

Today, with many effective treatments and associations available, the choice of first‐line therapy for CLL has never been more complex. All reimbursed treatments have demonstrated superiority over reference immunochemotherapy combinations; however, no head‐to‐head comparisons are available for first‐line CLL. Current recommendations are based on expert opinions, and the generation of real‐world data is important. To the best of our knowledge, this is the first real‐world study to investigate the criteria influencing the choice of first‐line treatment for CLL. During inclusion period, few patients were included in clinical trial making this analysis even more representative of “real‐life” practice, although, this low number of patients is somewhat close to what can be observed in solid oncology.^18^ Furthermore, the demographic characteristics of the patients observed in this study were similar to those reported in European and American epidemiological data.^19^, ^20^ Indeed, a median age of 72 years old, 61% of male subjects, 44% of *IGHV* mutated diseases, and 12% of *TP53* abnormalities reflects the general characteristics of a first‐line treatment population.

This study shows that the current CLL treatment decisions in France follow the recommended and published guidelines.^10^, ^11^, ^12^ Importantly, no patient received first‐line chemotherapy based on fludarabine, chlorambucil, or bendamustine. Immunotherapy consisting of anti‐CD20 antibody was used in three patients with CLL‐associated autoimmune cytopenia.

Regarding BTKis, the first important piece of information is the under‐prescription of ibrutinib compared with that of second‐generation BTKis. This likely reflects the tendency of prescribers to extrapolate evidence from relapsed settings to the first line, where second‐generation BTKis, acalabrutinib^21^ and zanubrutinib,^22^ have a better tolerability profile (ELEVATE‐RR and ALPINE studies, respectively). Our study highlights that BTKis are widely preferred by clinicians for elderly patients with unmutated *IGHV* status and *TP53* alterations. Considering the age criteria, the rationale behind this choice may be the opportunity to treat patients in an outpatient setting without the need for hospitalization and the fact that elderly people may not require a second‐line treatment after a continuous BTKi treatment, making the issue of potential resistance mutations less relevant; however, this argument was not included in the proposals concerning choice criteria in the questionnaire. Our data also suggest a predominant use of zanubrutinib prescription in patients with 17p deletions or *TP53* mutations. This observation emphasizes the importance of the data from the ALPINE trial comparing ibrutinib and zanubrutinib in the relapse setting, in which PFS was superior with zanubrutinib in patients with *TP53* alterations in a subgroup analysis^23^ and from the frontline SEQUOIA^24^ trial, the only trial with a dedicated arm (C arm) to patients with 17p deletion.^25^

Our results also show the predominant use of O‐Ven treatment, particularly in younger patients, those with fewer comorbidities, and those with a mutated *IGHV* status. The safety of the O‐Ven regimen in patients over 80 years of age has not been well described in the literature. The safety profile seems to be identical in the O‐Ven arms of the CLL13 and CLL14 studies dedicated to fit and unfit subjects, respectively.^26^ Nevertheless, “unfit” patients included in clinical trials are not representative of frail patients treated in daily clinical practice. Real‐life data on the tolerance and efficacy of Ven‐based regimens in patients over 80 years of age have been presented, showing that these combinations are feasible in selected patients.^27^ These results are consistent with the recommendations of academic societies, which all agree that given the excellent long‐term results of the CLL14 trial in this subset of patients, the O‐Ven combination is the reference treatment in the absence of *TP53* abnormalities and in cases of M‐*IGHV*.^26^

In this context, the role of the recently introduced combination of venetoclax and ibrutinib is difficult to define. Our data suggest its role in the specific case of bulky forms of CLL, where ibrutinib targets the tumoral aspect and the O‐Ven strategy seems to be less effective.^26^ In addition, these patients tend to be younger, which highlights that physicians are considering the cardiovascular toxicity of this strategy in unfit patients, as reported in the GLOW trial.^9^ Because the study period covered the first months of the availability of Ven‐I in France, it is possible that its current use is underestimated. It would therefore be interesting to reevaluate this aspect in the coming months after it has been available for a longer period of time.

The MCA and HCA showed two distinct patient clusters with differing therapeutic patterns. One of the main results was that ambulatory management, when possible, and a short and fixed treatment duration were variables of high importance in the treatment choice between the two clusters. These considerations illustrate the need for practical solutions that can be adapted to the constraints and concerns of patients' daily lives, especially as they get older. The choice of long‐term BTKi, despite its practicality, should not overshadow its possible adverse effects on elderly patients and the increasing reports of disease‐acquired mutations leading to increased treatment resistance due to long exposure. These patterns of patients should be taken into account for the development of CLL guidelines.

This study is a national study that could benefit from a larger international enrollment to consider national restrictions on the availability and reimbursement of certain treatments. The number of patients enrolled was significant, and most of the major French University hospitals involved in the French CLL cooperative group participated in the study. However, some are missing and could provide interesting additional data. Moreover, in France, more than 90% of CLL patients are treated in public centers. Regional centers are connected to academic centers through multidisciplinary meetings. Nevertheless, we cannot rule out the possibility that some patients were treated without first being presented at a multidisciplinary meeting and may therefore not be included in the study, representing a bias. Certain data were not collected, such as the Eastern Cooperative Oncology Group Performance Status assessment of the patient, which could provide additional information for the choice of treatment. In addition, treatment decision criteria were collected through physician‐completed questionnaires, which may expose the study to reporting or response bias. Although questionnaires were completed immediately after multidisciplinary team meetings and used standardized decision criteria across centers, some degree of subjectivity in physicians’ reporting cannot be fully excluded. Finally, as with all observational studies, causality cannot be inferred, and residual confounding cannot be excluded. Future research with a more comprehensive collection of clinical and genetic data, as well as broader international inclusion, could help validate these findings and enhance our understanding of treatment decision criteria.

In conclusion, this is the first study to explore real‐world therapeutic prescribing in the specific context of first‐line CLL in France, where five treatment strategies are available and recommended.

Our results revealed two contrasting patient profiles based on age, clinical characteristics, and genetic mutations that directly impacted clinician choice.

Second‐generation BTK inhibitors are widely used in elderly patients with unmutated *IGHV* status and logistical constraints, offering practical outpatient management solutions. In contrast, fixed‐duration treatment is widely preferred for younger patients, especially those with mutated *IGHV* status. These real‐life data underscore the importance of balancing clinical efficacy with patient‐specific factors, such as age, comorbidities, and treatment logistics, when selecting the optimal therapeutic approach for CLL.

## AUTHOR CONTRIBUTIONS

**Arthur Coste**: Investigation, writing–original draft, writing–review and editing, data curation, project administration, formal analysis, and resources. **Nanthara Sritharan**: Writing–review and editing and formal analysis. **Anne Lok**: Investigation and writing–review and editing. **Cécile Tomowiak**: Investigation and writing–review and editing. **Emmanuelle Ferrant**: Investigation and writing–review and editing. **Aline Clavert**: Investigation and writing–review and editing. **Damien Roos‐Weil**: Investigation and writing–review and editing. **Florian Bouclet**: Investigation and writing–review and editing. **Adrien Caillet**: Investigation and writing–review and editing. **Carolyne Croizier**: Investigation and writing–review and editing. **Nicolas Stocker**: Investigation and writing–review and editing. **Emmanuelle Tchernonog**: Investigation and writing–review and editing. **Pierre Feugier**: Investigation and writing–review and editing. **Alberto Santagostino**: Investigation and writing–review and editing. **Bénédicte Hivert**: Investigation and writing–review and editing. **Anne‐Sophie Michallet**: Investigation and writing–review and editing. **Sophie De Guibert**: Investigation and writing–review and editing. **Diane Lara**: Investigation and writing–review and editing. **Agathe Waultier‐Rascalou**: Investigation and writing–review and editing. **Fatiha Merabet**: Investigation and writing–review and editing. **Amandine Durand**: Investigation and writing–review and editing. **Lucile Bussot**: Investigation and writing–review and editing. **Kamel Laribi**: Investigation and writing–review and editing. **Marie‐sarah Dilhuydy**: Investigation, writing–review and editing, and conceptualization. **Vincent Lévy**: Investigation, conceptualization, methodology, writing–review and editing, writing–original draft, supervision, and validation. **Anne Quinquenel**: Conceptualization, investigation, methodology, writing–original draft, writing–review and editing, validation, and supervision.

## CONFLICT OF INTEREST STATEMENT

Florian Bouclet reports travel fees from AbbVie, BeiGene, Ltd, and Johnson and Johnson. Lucile Bussot reports fees for professional activities from AbbVie, AstraZeneca, and Johnson and Johnson. Adrien Caillet reports consulting fees from AbbVie, AstraZeneca, Beone, Johnson and Johnson, and Lilly. Sophie de Guibert reports consulting fees from AbbVie, AstraZeneca, BeiGene, Ltd, Johnson and Johnson, and Lilly. Marie Sarah Dilhuydy reports consulting fees from AbbVie, AstraZeneca, and Johnson and Johnson. Emmanuelle Ferrant reports consulting fees from AbbVie, BeiGene, Ltd, and Johnson and Johnson. Pierre Feugier reports consulting fees from Astra and Janssen Pharmaceuticals. Bénédicte Hivert reports fees for professional activities from AbbVie, BeiGene, Ltd, Bristol‐Myers Squibb, and Johnson and Johnson. Diane Lara reports honoraria from Novartis and Janssen Cilag; support for attending meetings and/or travel from Pfizer, Beigene, Janssen Cilag, and Amgen; and participation on a data safety monitoring board or advisory board from Beigene/Beone and Astra Zeneca. Kamel Laribi reports consulting fees from Janssen, Beigene, AbbVie, Takeda, Astra Zeneca, and Novartis; and grant and/or contract funding from Novartis, Beigene, AbbVie, and AstraZeneca. Vincent Lévy reports consulting fees from AbbVie, AstraZeneca, BeiGene, Ltd, CSL Behring, and Eli Lilly. Anne Lok reports consulting fees from Janssen Research & Development, LLC. Anne Quinquenel reports consulting fees from BeiGene, Ltd; fees for other professional activities from AstraZeneca, Johnson and Johnson, and Lilly; grant and/or contract funding from BeiGene, Ltd; and travel fees from AbbVie and Johnson and Johnson. Alberto Santagostino reports consulting fees from French Innovative Leukemia Organization (FILO). Emmanuelle Tchernonog reports consulting fees from AbbVie, BeiGene, Ltd, and Janssen Pharmaceuticals, Inc. Cécile Tomowiak reports consulting fees from AstraZeneca, BeiGene, Ltd, Johnson and Johnson, and Lilly; and travel fees from AbbVie, AstraZeneca, BeiGene, Ltd, Johnson and Johnson, and Lilly. The other authors declare no conflicts of interest.

## Supporting information

Supplementary Material S1

Figure S1

Figure S2

Figure S3

## Data Availability

The data that support the findings of this study are available from the corresponding author on reasonable request.
