# Supplementary material for: Factors influencing front line treatment of chronic lymphocytic leukemia: A French real‐world study
Source: Cancer. 2026 Apr 15;132(8):e70406. doi: 10.1002/cncr.70406 (PMC13082195; doi:10.1002/cncr.70406)
Supplement: Supplementary file 1 — Supplementary Material S1 [file CNCR-132-e70406-s001.docx]

**Supplemental data**

Supplemental Figure S1. Questionnaire filled by physicians

Supplemental Figure S2: MCA factorial plan with active modalities (green) and illustrative modalities (purple)

Supplemental Figure S3: comparison of academic and non-academic centers
