# Supplementary figures and images for: Factors influencing front line treatment of chronic lymphocytic leukemia: A French real‐world study
Source: Cancer. 2026 Apr 15;132(8):e70406. doi: 10.1002/cncr.70406 (PMC13082195; doi:10.1002/cncr.70406)

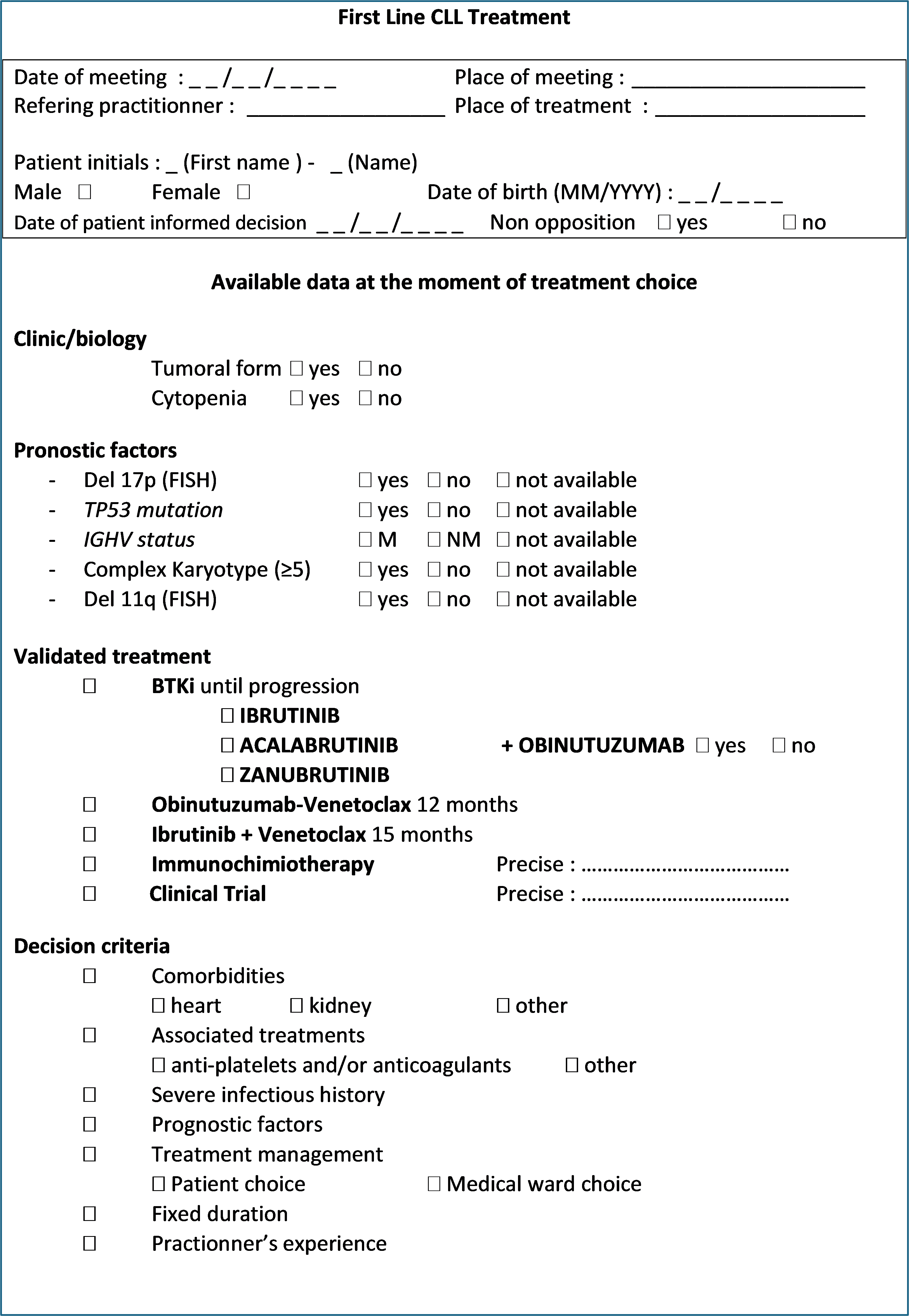

Supplement: Supplementary file 2 — Figure S1 [file CNCR-132-e70406-s004.png]

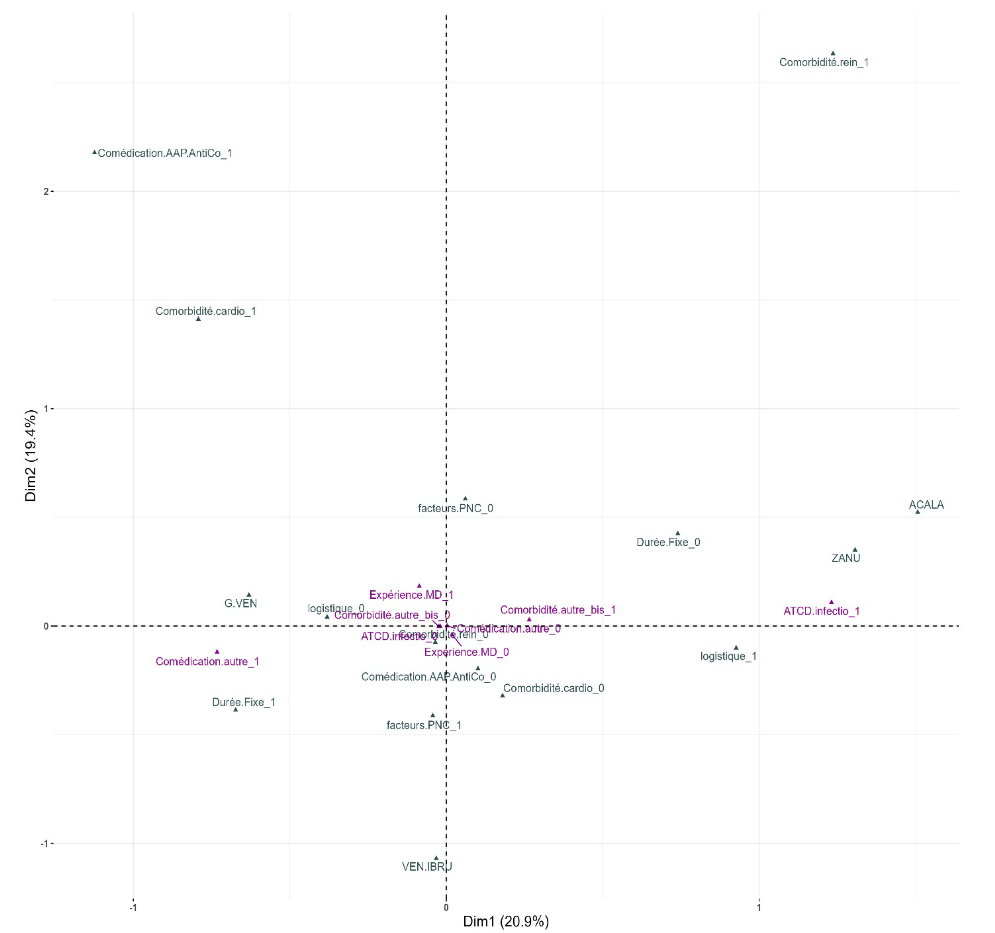

Supplement: Supplementary file 3 — Figure S2 [file CNCR-132-e70406-s003.png]

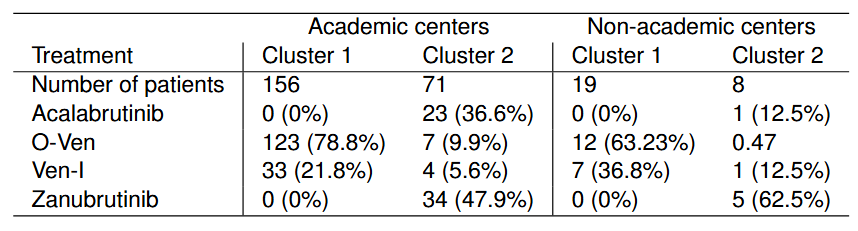

Supplement: Supplementary file 4 — Figure S3 [file CNCR-132-e70406-s002.PNG]
